# The Psychological Capital of Left-Behind University Students: A Description and Intervention Study From China

**DOI:** 10.3389/fpsyg.2018.02438

**Published:** 2018-12-05

**Authors:** Li Liang, Qianguo Xiao, Yisheng Yang

**Affiliations:** ^1^School of Psychology, Inner Mongolia Normal University, Hohhot, China; ^2^Psychological Development and Education Center, Southwest Petroleum University, Chengdu, China; ^3^Laboratory of Cognition and Mental Health, Chongqing University of Arts and Sciences, Chongqing, China

**Keywords:** university students, left-behind experience, psychological capital, group intervention, description study

## Abstract

The migrant worker phenomenon in China has negatively impacted the psychological development of these workers' children, whom researchers have termed “left-behind children” (LC) or university students with left-behind experience (USWL). Since USWL are the best among the LC in some sense, we decided to perform two investigations to determine if they might possess unique positive psychological capital factors. Study 1 aimed to explore the development of the psychological capital of USWL, and Study 2 utilized a group intervention design to improve USWL psychological capital. A questionnaire was administered to 281 USWL and 284 control university students in study 1. The results showed that the psychological capital of USWL was moderate, and their self-efficacy, optimism, hope, and overall psychological capital were significantly lower than those with no left-behind experience. However, their psychological resilience was remarkably higher than those who were not left behind. It also suggested that some demographic factors such as gender, grade, only child status, student leadership experience, reunion frequency with parents, and relationship with guardians significantly influence the psychological capital of USWL. In Study 2, a single-factor interventional experimental design based on the psychological capital intervention theory (PCI) was conducted in 73 USWL (38 in the experimental group, 35 in the control group). There were significant post-test differences between groups. Both the pre- and post-test results differed greatly in the experimental and control groups. Overall, our findings indicate that although the left-behind experience in childhood moderately impairs psychological capital development, it also fosters resilience. The psychological intervention based on PCI is an effective “remedy scheme” to improve their psychological capital qualities.

## Introduction

Left-behind children (LC) are juveniles whose parent(s) go out as migrant workers and therefore do not cohabitate with them. According to China's sixth census data from 2010, 6,102 children were left behind in China; among them, 37.7% were rural children, which accounted for 21.88% of the total number in the country (All-China Women's Federation, 2013). Their psychological development and mental health have been the focus of domestic researchers' attention. Several studies reported that these children experience psychological adjustment problems including loneliness (Hu et al., 2014), low self-esteem (Luo et al., 2012), depression and anxiety symptoms (He et al., 2012; Luo et al., 2012; Zhao et al., 2014; Wang et al., 2015), risk behaviors (Gao et al., 2010), and poor school performance and early dropout (Dreby, 2007; Wu et al., 2015; Fu et al., 2017). Studies have reported that migration type (father-only, mother-only, or both-parent migration) and care arrangements (Zhao et al., 2009; Jia and Tian, 2010; Graham and Jordan, 2011), as well as child age (Liu et al., 2009; Fan et al., 2010) and sex (Hu et al., 2014), are associated with the mental health outcomes of LC.

LC who matriculate to college have been termed university students with left-behind experience (USWL). It is significant to explore the effects of their unique experiences on psychological development. Li et al. (2017) reported that USWL have high levels of depression and anxiety and low levels of self-esteem and social support; they have more interpersonal problems and tend to be introverted. A study by Han et al. (2017) found that USWL are more likely to suffer negative events and have difficulty adapting to them; they have low gregariousness and unstable emotions including low self-esteem and loneliness. Several groups (Li and Liu, 2013; Xie, 2017; Zhao et al., 2017) concluded that the mental health level of USWL remained relatively lower than those without left-behind experience. However, some studies also described favorable effects of early left-behind experiences from the perspective of positive psychology. For example, USWL have much higher levels of volitional qualities; they are independent, optimistic, flexible, and good at learning and problem solving (Wen, 2011; Cao, 2017). In some aspects, the “left-behind experience” can be considered a gift rather than a psychological defect. From the positive psychology view, the left-behind experience may cultivate some important positive strengths that help improve individuals' adaptive behavior to cope with various problems.

There is no doubt that USWL face a longer period of unfavorable psychological development environment, and this has significant and wide-ranging impacts. They need more effective and powerful psychological resources to adapt, and developing resilience, one of the core Psychological Capital (PsyCap) factors, appears critically important. Resilience is an important psychological resource arising from human adaptation systems in the face of threats to development being those that compromise these protective systems (Wright and Masten, 2005). An individual's adaptive response to adverse events stems from their interactions with the environment and the processes that either promote well-being or protect against risk factors (Reich et al., 2010). In other words, a moderately unfavorable environment may offer better “soil” to cultivate one's resilience in the setting of cumulative “protective factors” (Russo and Stoykova, 2015). LC face some unfavorable circumstances including a lack of direct and timely care and support from their parents. However, their parent might become a migrant worker to provide their children with basic needs such as food and education. Therefore, the experience can provide some positive Psychological Capital or resources, especially for those LC who obtain academic success. Psychological Capital is defined as an individual's positive psychological state of development characterized by four core resources: (1) self-efficacy, (2) optimism, (3) hope, and (4) resiliency (Youssef and Luthans, 2007). This is an important set of psychological qualities that protect against adversity and pressure. Some studies found that PsyCap is closely related with individual psychological health, including self-esteem, internality, emotional balance, anxiety, depression, paranoia, and interpersonal sensitivity (Ouweneel et al., 2011; Krasikova et al., 2015). PsyCap represents positive resources that enable individuals to become successful (Sweetman and Luthans, 2010), and they can help university students meet academic challenges (Siu et al., 2014). In other words, PsyCap can buffer against unfavorable factors such as long-term absence of parents. In turn, the PsyCap of USWL can also be impacted by their unique experiences. We hypothesize that the psychological capital development of USWL is not seriously affected by the absence of their parents. In fact, they may develop prominent positive qualities such as independence and resilience (Wen, 2011; Cao, 2017). According to psychological capital intervention (PCI) theory, resilience activates cognitive, emotional, and behavioral processes that can change an individual's perception of his/her influence on their external conditions (Russo and Stoykova, 2015). If there are major adversities that undermine basic protective systems for development, it follows that efforts to promote competence and resilience in at-risk children should focus on strategies that protect or restore the efficacy of these basic systems. Resilience models and findings suggest that programs are most effective when they tap into these basic but powerful systems (Wright and Masten, 2005). Therefore, an exercise that visualizes and anticipates possible setbacks allows people to increase their ability to mentally re-frame those circumstances. The PCI theory was proposed by Luthans (Luthans and Youssef, 2004) to provide theoretical support and reference for interventions that develop psychological capital. This operational and feasible intervention method has four aspects of dimension: hope set-up, optimistic spirit cultivation, self-efficacy, and self-resilience enhancement. Studies have shown that intervention activities for young students can remarkably improve their psychological capital (Luthans et al., 2006b; Rew et al., 2014; Russo and Stoykova, 2015).

This study was developed to meet two main aims: (1) to understand psychological capital development among USWL and (2) to explore the effect of psychological intervention from the perspective of positive intervention. We hypothesize that: (1) USWL have lower overall psychological capital compared to those with no left-behind experience, but (2) they have higher levels of some dimensions of psychological capital such as resilience, and (3) a group intervention based on PCI theory can effectively improve USWL psychological capital.

## Study 1: The Development of the USWL' Psychological Capital

### Study 1 Method

#### Participants and Procedure

Cluster sampling was used to select 800 students from three universities in Sichuan Province, China, who voluntarily participated in the study. Among valid questionnaires, 726 were retrieved and identified 281 students with left-behind experience, 445 students who had no left-behind experience. Among the 281 students with left-behind experience, 148 were male and 133 were female; there were 150 freshmen, 78 sophomores, 23 juniors, and 30 seniors; 148 students were only children; and 67 were student leaders. The screening criterion for selecting USWL was individuals who had been taken care of by a single parent, other guardians, or themselves due to one or both parents working elsewhere for more than 6 months when they were between 0 and 16 years old. We randomly selected a control group of 284 students (141 male, 143 female) from the 445 students who had no left-behind experience. This study was carried out in accordance with the recommendations based on the guidelines by the Ethics Committees of Southwest Petroleum University, Sichuan Normal University, Xihua University in Sichuan Province in China. Written informed consent was obtained from all subjects.

### Measures

#### Demographic Questionnaire

We designed a questionnaire to gather data on gender, grade, number of siblings, student leadership experience, and left-behind experiences.

#### Positive Psychological Capital Questionnaire

The questionnaire (PCQ) was originally compiled by (Luthans et al., 2007), and translated into Chinese version by Li (see Luthans et al., 2006b, 2008). The questionnaire includes six items for each of the four factors of hope, optimism, efficacy, and resilience. However, PCQ is mainly applicable to staff and managers. The scope of its application is relatively limited, and there is still insufficient evidence of validity. Chinese scholar Zhang Kuo developed a more general, more applicable positive psychological capital questionnaire (PPQ) in 2010. In this study, we chose Zhang's et al. (2010) which using a 7-point Likert Scale with a total of 26 items including 4 dimensions showed higher reliability in Chinese samples. The scale used the Promax Skew Rotation method to get a 4-factor model, each factor had a eigenvalue >1, and the cumulative variance contribution rate was 54%. Among them, the variance contribution of factor 1 (Self-efficacy) was about 17%. Which including 7 items such as “My opinion and ability exceeds the average person” and “I am willing to take difficult and challenging work,” and the average of items commonality was 0.54. The variance contribution of factor 2 (Resilience) was about 14%. Which including 7 items such as “I can recover quickly when I encounter setbacks.” “A bad experience will make me depressed for a long time,” and the average of items commonality was 0.53. The variance contribution of factor 3 (Hope) was about 13%. Which including 6 items such as “I pursue my goals with confidence.” “I actively study and work to realize my ideals,” and the average of items commonality was 0.55. The variance contribution of factor 4 (Optimism) was about 10%, which including 6 items such as “I always see the good side of things.” “I think life is good,” and the average of items commonality was 0.52. The confirmatory factor analysis (CFA) of the four-factor model indicated a good fit to the data [*X*^2^/*df* = 1.50, root mean square error of approximation (RMSEA) = 0.049, comparative fit index (CFI) = 0.94, normed fit index (NFI) = 0.86, nonnormed fit index (NNFI) = 0.93, incremental fit index (IFI) = 0.94]. The scale used the Cronbach α coefficient as a measure of the internal consistency of the questionnaire. The α coefficients of the four sub-questionnaires were 0.86, 0.83, 0.80, and 0.76, respectively. The α coefficient of the full questionnaire was 0.90.

In this study, the internal consistency coefficient of the scale was 0.81, and the consistency coefficients for the self-efficacy, hope, optimism, and resilience subscales were 0.72, 0.76, 0.80, and 0.83, respectively. We tested the construct validity of PsyCap empirically with confirmatory factor analysis (CFA). The confirmatory factor analysis (CFA) of the four-factor model indicated a good fit to the data [*X*^2^/*df* = 1.54, root mean square error of approximation (RMSEA) = 0.042, comparative fit index (CFI) = 0.91, normed fit index (NFI) = 0.87, nonnormed fit index (NNFI) = 0.90, incremental fit index (IFI) = 0.90]

### Statistical Analyses

Descriptive statistics were calculated for the total psychological capital score of USWL. Student's *t*-tests were performed to compare psychological capital between USWL and the control group.

To compare demographic and experience characteristics, we performed *t*-tests (gender, only-child status, student leadership experience) or one-way analysis of variance (ANOVA) (grade, which parent left, left-behind duration, contact frequency with parents, reunion frequency with parents, guardian relationship).

Multiple regression analyses with the enter method were performed to further examine the influence of gender, grade, only-child status, student leadership experience, and left-behind experience on the psychological capital of USWL.

All data analyses were carried out with SPSS 20.0 software.

## Results

### Development Status of the Psychological Capital of USWL

The total score of the psychological capital of USWL was at a moderate level (4 points above the medium critical value). In view of each factor, the average four-factor score was higher than the middle of the critical value. Overall, USWL PsyCap was well.

Statistical analyses showed that the self-efficacy, optimism, hope, and PsyCap of university students who were not being left-behind in childhood were significantly higher than those of USWL (respectively: *df* = 563, *t* = −5.41, *p* < 0.001; *df* = 563, *t* = −6.61, *p* < 0.001; *df* = 563, *t* = −5.61, *p* < 0.01; *df* = 563, *t* = −3.03, *p* < 0.01). However, resilience of USWL was significantly higher compared to that of the control group (*df* = 563, *t* = 10.31, *p* < 0.01). Specific results are shown in Table 1.

**Table 1:** Comparison of psychological capital to the university students who were left-behind or not.

**Factors**	**Self-efficacy**	**Optimism**	**Hope**	**Resilience**	**PsyCap**
Left-behind (*n* = 281)	4.10 ± 0.63	4.28 ± 0.64	4.44 ± 0.67	4.87 ± 0.71	4.41 ± 0.45
None left-behind (*n* = 284)	4.43 ± 0.84	4.72 ± 0.92	4.85 ± 1.04	4.18 ± 0.75	4.54 ± 0.63
*t*	−5.41^***^	−6.61^***^	−5.61^***^	10.31^***^	−3.03^**^
*Cohen*'*s d*	0.44	0.56	0.47	0.94	0.24
*Effect Size*	0.22	0.27	0.23	0.43	0.12

^*^ p < 0.05;

** *p < 0.01*;

*** *p < 0.001. PsyCap, psychology capital*.

### Impacts of Gender, Grade, Only-Child Status, and Student Leader Experience

The association of psychological capital of USWL with gender, grade, only-child status, and student leader experience was determined. The results revealed significant differences in some dimensions of psychological capital in view of the factors above. Specifically, boys' psychological capital, self-efficacy, and resilience were significantly higher than that of girls (*df* = 279, *t* = 3.77, *p* < 0.001; *df* = 279, *t* = 4.18, *p* < 0.001; *df* = 279, *t* = 2.84, *p* < 0.01); students with siblings had significantly higher hope than only children (*df* = 279, *t* = 2.17, *p* < 0.05). In other dimensions of psychological capital, students with siblings scored higher than only children, but the differences were not statistically significant. USWL with leadership experience also had greater hope (*df* = 279, *t* = 2.17, *p* < 0.05). Left-behind students in different grades showed differences in total psychological capital scores and dimensions of self-efficacy, hope, and resilience (*df* = 277, *F* = 11.21, *p* < 0.001; *df* = 277, *F* = 6.92, *p* < 0.01; *df* = 277, *F* = 8.39, *p* < 0.001; *df* = 277, *F* = 8.96, *p* < 0.001). Details are in Tables 2, 3.

**Table 2:** Demographic differences in USWL psychological capital.

**Factors**		**Self-efficacy**	**Optimism**	**Hope**	**Resilience**	**PsyCap**
Gender	Male	4.25 ± 0.70	4.50 ± 0.70	4.34 ± 0.66	4.92 ± 0.65	4.44 ± 0.49
	Female	3.94 ± 0.51	4.37 ± 0.62	4.22 ± 0.62	4.69 ± 0.76	4.40 ± 0.40
	*t*	4.18^***^	1.70	1.54	2.84^**^	3.77^***^
	*Cohen*'*s d*	0.51	0.20	0.19	0.33	0.09
	*Effect Size*	0.25	0.10	0.09	0.16	0.04
Only-child status	Yes	4.06 ± 0.64	4.46 ± 0.63	4.19 ± 0.60	4.77 ± 0.72	4.37 ± 0.41
	No	4.13 ± 0.63	4.43 ± 0.70	4.36 ± 0.67	4.85 ± 0.70	4.44 ± 0.49
	*t*	0.89	−0.37	2.17^*^	−0.96	1.32
	*Cohen*'*s d*	0.11	0.05	0.27	0.11	0.15
	*Effect Size*	0.06	0.02	0.13	0.06	0.07
Student leader experience	Yes	4.12 ± 0.57	4.49 ± 0.65	4.44 ± 0.60	4.90 ± 0.70	4.49 ± 0.45
	No	4.09 ± 0.65	4.43 ± 0.67	4.23 ± 0.64	4.78 ± 0.71	4.38 ± 0.45
	*t*	0.35	0.66	2.48^*^	1.16	1.67
	*Cohen*'*s d*	0.05	0.09	0.34	0.17	0.24
	*Effect Size*	0.02	0.05	0.17	0.08	0.12

* *p < 0.05*;

** *p < 0.01*;

*** *p < 0.001. PsyCap, psychology capital*.

**Table 3:** Comparison on PsyCap results of USWL in different grades factors.

**Factors**	**Self-efficacy**	**Optimism**	**Hope**	**Resilience**	**PsyCap**
Freshman (*n* = 150)	4.20 ± 0.55	4.28 ± 0.64	4.44 ± 0.67	4.87 ± 0.71	4.41 ± 0.45
Sophomore (*n* = 78)	3.83 ± 0.76	4.72 ± 0.92	4.85 ± 1.04	4.18 ± 0.75	4.54 ± 0.63
Junior (*n* = 23)	4.17 ± 0.56	4.06 ± 0.71	4.48 ± 0.51	4.95 ± 0.53	4.41 ± 0.32
Senior (*n* = 30)	4.25 ± 0.56	4.21 ± 0.55	4.23 ± 0.74	4.94 ± 0.72	4.41 ± 0.53
*F*	6.92^***^	8.39^***^	2.35	8.96^***^	11.21^***^
*η^2^*	0.07	0.08	0.03	0.09	0.11
*Post-hoc*	Freshman > Sophomore Senior > Sophomore	Freshman > Sophomore Freshman > Junior	-	Freshman > Sophomore Junior > Sophomore Senior > Sophomore	Freshman > Sophomore

^*^ p < 0.05;

^**^ p < 0.01;

*** *p < 0.001. PsyCap, psychology capital*.

ANOVAs were performed to investigate the psychological capital of USWL in different left-behind experiences (parents who went out working, left-behind duration, frequency of contacting and reuniting with their parents, relationships with guardians). The results showed that parents who went out working and left-behind duration affected the psychological capital of USWL, as well as self-efficacy, hope, optimism, and resilience. Significant differences in the total psychological capital score and other dimensions depended on the different frequency that the LC contacted with their parents, except for the implicit score of resilience (*df* = 276, *F* = 1.46, *p* > 0.05). Students who contacted their parents every 1 or 2 weeks had significantly higher self-efficacy, hope, and optimism and a higher total score of psychological capital than those who contacted their parents once a year or less frequently (*df* = 276, *F* = 6.47, *p* < 0.001; *df* = 276, *F* = 8.00, *p* < 0.001; *df* = 276, *F* = 3.99, *p* < 0.001; *df* = 276, *F* = 7.79, *p* < 0.001). During the separation, the psychological capital and every dimension of USWL varied with the frequency of contact. Subjects who reunited each month had significantly higher self-efficacy, hope, optimism, and psychological capital scores than those who reunited once a year. However, USWL who had one-time contact with their parents every year had the highest resilience scores, while those with monthly reunions students had lowest (*df* = 276, *F* = 6.14, *p* < 0.001). Guardian relationship also affected the score of the psychological capital for the hope and optimism dimensions. USWL whose grandparents cared for them usually scored higher in hope, optimism, and total psychological capital score than those whose guardians were other relatives or neighbors (*df* = 276, *F* = 5.50, *p* < 0.001; *df* = 276, *F* = 3.75, *p* < 0.01; *df* = 276, *F* = 3.00, *p* < 0.01). Details are presented in Table 4.

**Table 4:** Comparison on psychological capital of USWL with different left-behind Experiences.

		**Self-efficacy**	**Optimism**	**Hope**	**Resilience**	**PsyCap**
Parents who went out working	Both	4.07 ± 0.66	4.43 ± 0.71	4.24 ± 0.70	4.76 ± 0.67	4.38 ± 0.48
	Father	4.13 ± 0.58	4.45 ± 0.63	4.30 ± 0.58	4.86 ± 0.74	4.44 ± 0.40
	Mother	4.27 ± 0.77	4.42 ± 0.47	4.56 ± 0.18	4.97 ± 0.92	4.55 ± 0.47
	*F*	0.85	0.04	1.64	1.03	1.33
	*η^2^*	0.07	0.00	0.01	0.01	0.01
Left-behind duration (year)	0.5–1	4.31 ± 0.69	4.55 ± 0.64	4.31 ± 0.64	4.97 ± 0.63	4.47 ± 0.45
	1–3	4.10 ± 0.56	4.39 ± 0.64	4.28 ± 0.69	4.92 ± 0.63	4.42 ± 0.43
	3–5	4.09 ± 0.67	4.39 ± 0.73	4.26 ± 0.53	4.74 ± 0.75	4.41 ± 0.45
	5 or more	3.97 ± 0.67	4.36 ± 0.65	4.25 ± 0.66	4.69 ± 0.77	4.33 ± 0.50
	*F*	2.49	1.39	0.13	2.23	0.92
	*η^2^*	0.03	0.02	0.00	0.02	0.01
Frequency of contacting with parents (month)	1/4–1/2	4.24 ± 0.56	4.54 ± 0.60	4.42 ± 0.52	5.10 ± 0.60	4.51 ± 0.36
	1–3	3.90 ± 0.71	4.53 ± 0.53	4.42 ± 0.67	4.87 ± 0.74	4.47 ± 0.19
	6	3.86 ± 0.71	4.44 ± 0.67	4.03 ± 0.70	4.83 ± 0.74	4.31 ± 0.49
	12 or more	3.81 ± 0.41	4.17 ± 0.91	3.96 ± 0.76	4.68 ± 0.82	4.17 ± 0.60
	*F*	6.47^***^	3.99^***^	8.00^***^	1.46	7.79^***^
	*η^2^*	0.09	0.06	0.10	0.02	0.10
Frequency of reuniting with parents (times/year)	0	4.31 ± 0.61	4.54 ± 0.60	4.42 ± 0.52	5.10 ± 0.60	4.51 ± 0.36
	1	4.25 ± 0.68	4.53 ± 0.53	4.42 ± 0.67	4.87 ± 0.74	4.47 ± 0.19
	2	4.02 ± 0.62	4.44 ± 0.67	4.03 ± 0.70	4.83 ± 0.74	4.31 ± 0.49
	3–4	4.01 ± 0.46	4.17 ± 0.91	3.96 ± 0.76	4.68 ± 0.82	4.17 ± 0.60
	12	3.98 ± 0.64	4.17 ± 0.91	3.96 ± 0.76	4.68 ± 0.82	4.17 ± 0.60
	*F*	2.85^*^	4.00^**^	3.56^**^	6.14^***^	4.16^**^
	*η^2^*	0.04	0.06	0.05	0.08	0.06
Relationships with guardians	Grandparents	4.19 ± 0.66	4.50 ± 0.58	4.86 ± 0.82	4.84 ± 0.72	4.51 ± 0.66
	Father or Mother	4.19 ± 0.50	4.48 ± 0.67	4.28 ± 0.89	4.84 ± 0.48	4.42 ± 0.44
	Relatives or Neighbors	3.71 ± 0.87	4.44 ± 0.67	3.70 ± 1.01	4.83 ± 0.77	3.95 ± 0.83
	Sibling	4.19 ± 0.66	3.83 ± 1.02	4.25 ± 0.61	4.55 ± 0.43	4.42 ± 0.41
	Own	3.71 ± 0.87	4.17 ± 0.35	4.24 ± 0.47	4.56 ± 0.83	4.36 ± 0.22
	*F*	1.30	3.75^**^	5.50^***^	0.90	3.00^**^
	*η^2^*	0.02	0.05	0.07	0.01	0.04

* *p < 0.05*;

** *p < 0.01*;

*** *p < 0.001. PsyCap, psychology capital*.

We performed multiple regression analysis with the enter method to further examine the influence of gender, grade, only-child status, student leadership experience, and left-behind experiences (including which parents went out working, left-behind duration, contact and reunion frequencies, and guardian relationships) on the psychological capital of USWL. Gender, only-child status, and leadership experience were coded as 0 or 1: Male code 1, female code 0; only child code 1, non-only child code 0; as a student cadre code 1, not as a student cadre code 0; Since there are 4 levels in the grade, three virtual grade variables (Sophomore/Freshman, Junior/Freshman, Senior/Freshman) were established with the Freshmen as level 1. Two virtual “Parents who went out working” variables were used (only the father/Both, only the mother/both), with both parents as level 1. There were three virtual “left-behind duration” variables (1–3 years and 0.5–1 year, 3–5 years and 0.5–1 year, 5 years or more and 0.5–1 year), with 0.5–1 year as level 1. Three virtual “Contact Frequency with Parent” variables (1–3 months and 1/4–1/2 month, 6 months and 1/4–1/2 month, 12 months and 1/4–1/2 months) were established with 1/4–1/2 months as level 1. There were four virtual “Reunion Frequency with Parents” variables (1 time/year and 0 times/year, 2 times/year and 0 times/year, 3–4 times/year & 0 times/year, 12 times/year and 0 times/year) established with the 0 times/year as level 1. There were four virtual “Guardian” variables (father or mother/grandparents, relatives/neighbors/grandparents, siblings/grandparents, self/grandparents) established with grandparents considered level 1.

Based on preliminary results, all variables except for “only the father went out working” were entered in the regression equation for total Psychological Capital and its dimensions. Self-efficacy: *R* = 0.504, *R*^2^ = 0.254, *R*^2^*adj* = 0.194, *F* = 4.21(*P* < 0.001); Optimism: *R* = 0.439, *R*^2^ = 0.193, *R*^2^*adj* = 0.128, *F* = 2.95(*P* < 0.001); Hope: *R* = 0.561, *R*^2^ = 0.315, *R*^2^*adj* = 0.259, *F* = 5.67(*P* < 0.001); Resilience: *R* = 0.495, *R*^2^ = 0.245, *R*^2^*adj* = 0.184, *F* = 4.00(*P* < 0.001); PsyCap: *R* = 0.571, *R*^2^ = 0.326, *R*^2^*adj* = 0.272, *F* = 5.98 (*P* < 0.001). The results shown in Table 5 indicate that these variables can jointly predict the variance of 25.4% for self-efficacy; 31.5% for hope, 19.3% for optimism, and 32.6% for PsyCap. Among these variables, gender has a significant predictive effect on self-efficacy, optimism, hope, resilience and PsyCap. Grade has a significant predictive effect on Hope. Student leadership experience has significant predictive effects for hope and PsyCap.

**Table 5:** Multiple regression analysis on psychological capital of USWL (*n* = 218).

	**Self-efficacy**				**Optimism**				**Hope**				**Resilience**				**PsyCap**			
	**B**	**SE**	***t***	***P***	**B**	**SE**	***T***	***P***	**B**	**SE**	***t***	***P***	**B**	**SE**	***t***	***P***	**B**	**SE**	***t***	***P***
Gender	0.30	0.08	3.96	0.000	0.20	0.08	2.49	0.01	0.08	0.07	1.12	0.26	0.27	0.08	3.24	0.001	0.21	0.05	4.20	0.000
Only-child status	−0.07	0.07	−0.89	0.38	−0.09	0.08	−1.12	0.26	0.13	0.07	1.85	0.07	−0.04	0.08	−0.51	0.61	−0.02	0.05	−0.33	0.74
Student leadership experience	0.03	0.09	0.30	0.77	0.10	0.10	1.00	0.32	0.27	0.09	3.09	0.002	0.15	0.10	1.50	0.14	0.14	0.06	2.23	0.03
**GRADE**																				
Sophomore and freshman	−0.34	0.09	−4.00	0.000	−0.23	0.09	−2.46	0.02	−0.42	0.08	−5.09	0.000	−0.44	0.10	−4.53	.000	−0.36	0.06	−6.08	0.000
Junior and freshman	0.09	0.14	0.65	0.52	−0.03	0.15	−0.21	0.83	−0.43	0.13	−3.24	0.001	−0.04	0.15	−0.28	0.78	−0.10	0.09	−1.09	0.28
Senior and freshman	−0.02	0.13	−0.15	0.88	−0.34	0.14	−2.34	0.02	−0.38	0.13	−3.01	0.003	−0.14	0.15	−0.94	0.35	−0.22	0.09	−2.471	0.01
**PARENTS WENT OUT WORKING**																				
Only the father and Both	–	–	–	–	–	–	–	–	–	–	–	–	–	–	–	–	–	–	–	–
Only the mother and Both	−0.09	0.09	−0.91	0.36	0.09	0.10	0.87	0.38	−0.13	0.09	−1.46	0.15	−0.05	0.11	−0.44	0.66	−0.04	0.06	−0.70	0.49
**LEFT-BEHIND DURATION**																				
1–3 years and 0.5–1 year	−0.05	0.10	−0.52	0.61	−0.07	0.11	−0.68	0.50	0.06	0.10	0.61	0.54	0.01	0.11	0.12	0.91	−0.01	0.07	−0.20	0.84
3–5 years and 0.5–1 year	0.25	0.11	2.26	0.02	−0.13	0.12	−1.08	0.28	0.01	0.11	0.11	0.91	0.32	0.12	2.55	0.01	0.11	0.08	1.49	0.14
5 years or more and 0.5–1 year	0.05	0.10	0.50	0.62	−0.20	00.11	−1.86	0.07	−0.08	0.09	−0.83	0.41	0.26	0.11	2.39	0.02	0.01	0.07	0.13	0.89
**CONTACT FREQUENCY**																				
1–3 months and 1/4–1/2 months	−0.32	0.09	−3.63	0.000	−0.27	0.10	−2.86	0.01	−0.43	0.09	−5.00	0.000	−0.07	0.10	−0.74	0.46	−0.30	0.17	−1.76	0.08
6 months and 1/4–1/2 months	−0.47	0.14	−3.42	0.001	0.15	0.15	1.02	0.31	−0.38	0.13	−2.84	0.01	−0.10	0.16	−0.66	0.51	−0.23	0.19	−1.23	0.22
12 months and 1/4–1/2 months	−0.18	0.24	−0.77	0.44	0.06	0.26	0.24	0.81	0.17	0.23	0.73	0.46	0.28	0.27	1.05	0.30	0.05	0.23	0.24	0.81
**REUNION FREQUENCY**																				
1 time/year and 0 time/year	0.05	0.11	0.46	0.65	0.33	0.12	2.86	0.01	0.30	0.10	2.91	0.00	−0.04	0.12	−0.35	0.73	0.05	0.23	−2.18	0.03
2 time/year and 0 time/year	0.28	0.12	2.37	0.02	0.18	0.13	1.43	0.16	0.18	0.11	1.59	0.11	0.28	0.13	2.10	0.04	−0.16	0.07	1.00	.32
3–4 times/year and 0 time/year	0.10	0.15	0.68	0.50	0.13	0.16	0.77	0.44	0.05	0.15	0.31	0.76	0.58	0.17	3.40	0.00	0.07	0.07	0.59	0.56
12 times/year and 0 time/year	0.17	0.15	1.17	0.25	0.44	0.16	2.77	0.01	0.25	0.06	0.14	0.44	0.34	0.17	2.06	0.04	0.05	0.09	1.07	0.29
**RELATIONSHIPS WITH GUARDIANS**																				
Father or mother and grandparents	0.07	0.10	0.66	0.51	−0.02	0.11	−0.19	0.85	0.51	−0.08	0.10	−0.83	0.41	0.11	−0.50	0.62	−0.02	0.07	−0.32	0.75
Relatives/neighbors and grandparents	0.09	0.14	0.67	0.50	−0.45	0.15	−3.04	0.003	0.50	−0.10	0.13	−0.80	0.43	0.15	−0.44	0.66	−0.13	0.09	−1.42	0.16
Siblings and grandparents	−0.11	0.20	−0.53	0.59	−0.41	0.21	−1.89	0.06	0.59	−0.26	0.19	−1.36	0.18	0.22	−0.95	0.34	−0.25	0.14	−1.82	0.07
Own and grandparents	0.15	0.16	0.93	0.35	−0.11	0.17	−0.61	0.54	0.35	0.46	0.15	2.97	0.003	0.18	−1.95	0.05	0.03	0.11	0.28	0.78

In study 1, we found the total psychological capital of USWL was moderate, and their self-efficacy, optimism, hope, and overall psychological capital were significantly lower than those with no left-behind experience. This result showed that there was a relatively large space for the USWL' psychological capital development. Therefore, we designed a group intervention program to increase their psychological capital, and adopted a single-factor interventional experiment to verify the effectiveness of the intervention program (study 2). In study 2, we aimed to develop a simple but effective intervention program to improve Chinese USWL'PsyCap.

## Study 2: USWL Psychological Intervention

Flyers describing the study were posted in public places, and 100 USWL at a university in Sichuan Province were recruited. Recruitment was voluntary. Subjects were informed about the purpose, process, and duration of the experiment. They then completed the positive psychological capital questionnaire. We selected 73 USWL who had lower scores in the psychological capital questionnaire, and these subjects were randomly divided into experimental (*n* = 38) and control (*n* = 35) groups. This study was approved by the Psychology Ethics Committee, and all participants provided written informed consent prior to participation.

## Study 2 Methods

### Positive Psychological Capital Questionnaire (as in Study 1)

#### Self-Designed Group Activity Effect Questionnaire

We referenced the relative content of the Group Psychological Consultation by Fan Fuming and combined it with group counseling (Fan, 2005). Students were asked to make comments on four aspects: participation, trust, attainment, transfer, and application. We also included two open items: What impressed you the most in the activity? What is the most important thing you learned? There were nine quantitative questions scored on a 5-level Likert scale: 1 point for strongly disagree, 2 for disagree, 3 for neither agree nor disagree, 4 for agree, 5 for strongly agree. The scale used the Cronbach α coefficient as a measure of the internal consistency of the questionnaire. The α coefficients of the questionnaire items ranged from 0.69 to 0.83, and the total questionnaire value was 0.87. The correlations between questionnaire dimensions were 0.18–0.57; and the correlations between the dimensions and the total questionnaire were 0.62–0.83. These values demonstrate that the questionnaire was constructively valid.

### Theoretical Basis of the PCI

This study took Luthans' psychological capital theory and intervention model (Luthans and Youssef, 2004) as an important theoretical basis. The PCI model consists of four levels. First, plans are made to develop hope by planning goals and ways to achieve them. Secondly, accept limitations to strengthen belief and accumulate experience, developing optimism. Third, develop self-efficacy by inspiring students to experience success. Finally, take advantage of effective resources and interpersonal relations to develop resilience.

### Group Intervention Plan

Based on PCI theory, a specific psychological capital group intervention was developed considering USWL characteristics and the four dimensions of psychological capital. There were eight themes in total, each comprising a warm-up, theme activities, and concluding activity. Theme 1: We are family. Goal: Students shall form a group family and become part of it, developing a sense of trust and belonging. Family members discuss their group contracts and norms. Theme 2: Invest trust. Goal: Strengthen trust, belonging, and team cohesion among group members. Theme 3: Self-exploration. I am capable. Goal: Guide members to understand themselves comprehensively and objectively, accept their limitations, and learn reasonable attributions to enhance self-efficacy and self-esteem. Theme 4: Release emotions and try to master them. Goal: Find out where the emotion stems from and try to use ABC emotional theory to control emotions and to form an optimistic explanatory style. Theme 5: Defuse the stress with optimistic spirit. Goal: Stimulate a stress experience and try to overcome it with relaxation and problem solving. Theme 6: Laugh at setbacks. Strengthen resilience. Goal: Develop patience and perseverance to strengthen endurance and resilience. Theme 7: Cultivate hope, look forward to the future. Goal: Help group members set reasonable goals and explore ways to achieve them, increasing hope. Theme 8: Sharing. Conclude the intervention. Goal: Review and summarize the group experience, discuss mutual responses, and say goodbye. Apply the integration of resources and strength gained from the intervention to support group members.

### Experimental Design and Procedures

**Figure 1:**
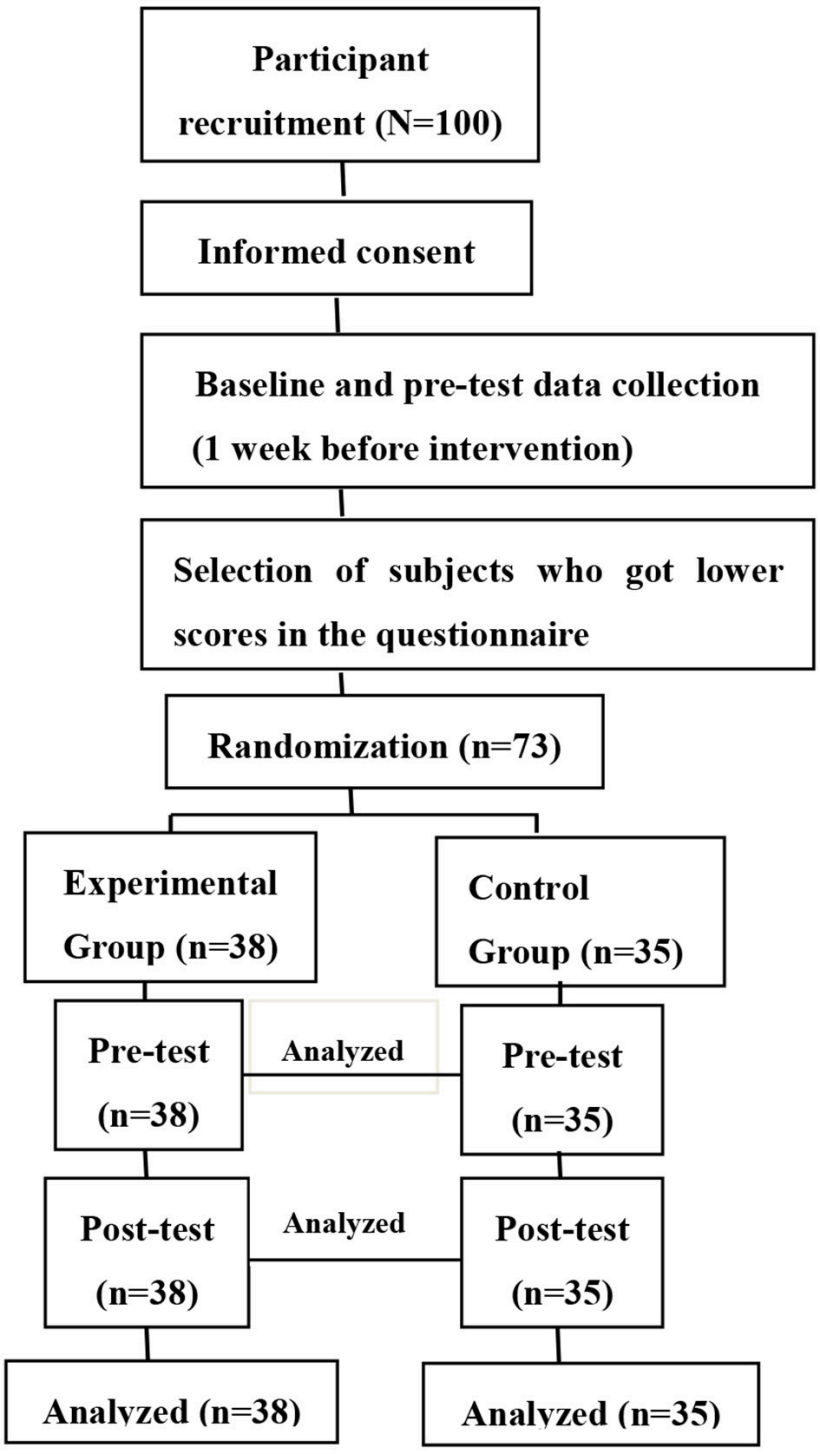
Flow of the experimental study with a randomized pre-post controlled design.

The study was based on a single-factor experiment with group psychological intervention as the independent variable. The dependent variables were the pre- and post-test scores of the psychological capital scale (i.e., self-confidence, optimism, hope, and resilience). The research was divided into three stages. Stage 1: Pre-test. Subjects in the experimental and control groups completed the psychological capital questionnaire before the group intervention. Stage 2: The author of this study administered the intervention for USWL through self-designed psychological capital intervention plans. Once-weekly counseling for 1.5 h each was provided for 8 weeks in total. Stage 3: Post-test. At the end of group activities, both groups completed the psychological capital test and a self-assessment of the group psychological counseling effect scale. Figure 1 summarizes the flow of the study.

### Statistical Analysis

A single-factor experimental design based on PCI was conducted following group intervention. Student's *t*-tests were performed to compare Psychological Capital between the experimental and control groups before the intervention, within groups before and after the intervention, and between groups after the intervention. All data analyses were carried out with SPSS 20.0 software.

## Research Results and Analysis

### PsyCap at Pretest

The descriptive results of USWL' PsyCap at pretest were shown in Table 6. One-way analysis of variance (ANOVA) revealed that there were no significant gender, grade, number of siblings, student leadership experience, and left-behind experiences differences on PsyCap (ps > 0.10). Then comparison between the experimental intervention and control group before the intervention, Table 7 shows that there were no significant differences between the two groups with regard to the total score of psychological capital (*t* = 0.83, *p* = 0.41, Cohen's d = 0.20, Effect Size = 0.10) and the four dimensions (Self-efficacy: *t* = 0.58, *p* = 0.56, Cohen's d = 0.14, Effect Size = 0.07; Resilience: *t* = 0.75, *p* = 0.45, Cohen's d = 0.18, Effect Size = 0.09; Hope: *t* = 0.42, *p* = 0.68, Cohen's *d* = 0.10, Effect Size = 0.05; Optimism: *t* = 0.55, *p* = 0.59, Cohen's d = 0.13, Effect Size = 0.07).

**Table 6:** Descriptive results of USWL' PsyCap at pretest (M ± SD).

	**Self-efficacy**	**Resilience**	**Hope**	**Optimism**	**PsyCap**
Number of items	7	7	6	6	26
Maximum value	7.00	7.00	7.00	7.00	6.60
Minimum value	1.00	1.00	1.00	1.00	2.18
M ± SD	4.04 ± 1.04	4.38 ± 1.15	4.54 ± 1.20	4.66 ± 1.19	4.41 ± 0.91

**Table 7:** PsyCap of two groups at different test sessions (M ± SD).

**Factors**		**Self-efficacy**	**Optimism**	**Hope**	**Resilience**	**PsyCap**
Experimental group (*n* = 38)	Pre-test	3.67 ± 1.26	4.26 ± 1.23	4.07 ± 1.15	4.14 ± 1.10	4.04 ± 0.84
	Post-test	5.20 ± 0.41	5.91 ± 0.68	5.77 ± 0.81	5.20 ± 1.20	5.52 ± 0.58
	*T*	−7.10^**^	−7.23	−7.42	−3.98	−8.97
	*Cohen*'*s d*	−1.63	−1.66	−1.71	−0.92	−2.05
	*Effect Size*	−0.63	−0.64	−0.65	−0.42	−0.72
Control group (*n* = 35)	Pre-test	3.53 ± 0.73	4.12 ± 0.81	3.97 ± 0.94	3.97 ± 0.81	3.90 ± 0.53
	Post-test	3.68 ± 0.34	4.27 ± 0.90	4.30 ± 0.88	4.15 ± 1.02	4.10 ± 0.45
	*t*	−1.13	−0.72	−1.49	−0.82	−1.71
	*Cohen*'*s d*	−0.26	−0.18	−0.36	−0.20	−0.41
	*Effect Size*	−0.13	−0.10	−0.18	−0.09	−0.20

^*^ p < 0.05;

** *p < 0.01*;

^***^ p < 0.001. PsyCap, psychology capital.

### Effect of Intervention on PsyCap

The results of PsyCap for the experimental intervention and control groups are displayed in Table 7. At the pretest session, there was no significant difference in the level of PsyCap and its four factors (self-efficacy, resilience, hope, optimism) between the experimental intervention group and the control group (*t* = 0.83, *p* = 0.41, Cohen's *d* = 0.20, Effect Size = 0.10; *t* = 0.58, *p* = 0.56, Cohen's *d* = 0.14, Effect Size = 0.07; *t* = 0.75, *p* = 0.45, Cohen's *d* = 0.18, Effect Size = 0.09; *t* = 0.42, *p* = 0.68, Cohen's *d* = 0.10, Effect Size = 0.05; *t* = 0.55, *p* = 0.59, Cohen's *d* = 0.13, Effect Size = 0.07), which indicates the success of the random assignment of participants.

### Post-test Comparisons

Both groups completed the psychological capital questionnaire after the psychological group intervention. We observed remarkable differences between the two groups. The total scores for psychological capital (*t* = 11.56, *p* = 0.000, Cohen's *d* = 2.73, Effect Size = 0.81) and the four dimensions were remarkably higher in the experimental group (Self-efficacy: *t* = 17.00, *p* = 0.000, Cohen's *d* = 4.04, Effect Size = 0.90; Resilience: *t* = 3.98, *p* = 0.000, Cohen's *d* = 0.94, Effect Size = 0.43; Hope: *t* = 7.45, *p* = 0.000, Cohen's d = 1.74, Effect Size = 0.66; Optimism: *t* = 8.83, *p* = 0.000, Cohen's *d* = 2.06, Effect Size = 0.72), indicating that intervention can effectively improve the psychological capital.

### Analysis of the Group Intervention

A self-designed group activity feedback questionnaire was administered to the experimental group after the intervention. A large majority (94.7%) of subjects in the experiment group took the activity seriously, 89.5% felt satisfied with it, 92.1% were satisfied with the instructor, and 94.7% were satisfied with the whole process. Among them, 92.1% felt they benefitted from the activity, and 94.7% experienced self-development. Negative effects were reported by 5.3% of subjects. Importantly, 95% of the participants in the experimental group felt more confident. They realized their sense of inferiority could actually inspire self-realization. Feedback from students included statements like, “I felt inferior since I was a child for I came from countryside and was left behind. When I got into the university, this became even worse. I saw excellent classmates who made me more nervous and cowardly. I felt like I was garbage. But during this activity, I found that we have something in common, and being left behind is no big deal. We deserve to be treated well.” There were 90% of students who thought that group counseling helped them take a second look at their growth experience and discover positive power from it. They treasure life and enjoy developing friendships. Statements included, “I want to live positively. There are always negative things in life, it depends on how we face it” and “We are left behind, but I am not alone. Complain and resentment changes nothing. I am going to accept the fact and embrace it.”

## Discussion

### Discussion of Study 1

The results of study 1 showed that the total psychological capital of USWL was higher than the middle critical value of 3.5, which was at a moderate level. In view of the single factor, the average scores of the four factors were higher than the middle critical value. The scores from highest to lowest were for resilience, hope, optimism, and self-efficacy. Overall, USWL had well-developed psychological capital. This is consistent with the findings of previous studies (Luo et al., 2012; Li and Xin, 2015). It supports our hypothesis that left-behind experience in childhood does not necessarily lead to unhealthy mental development.

Notably, USWL resilience was remarkably higher than that of university students with no left-behind experience. Some studies reported no differences in certain mental health outcomes (e.g., school satisfaction and happiness) between left-behind and control children (Hu et al., 2008). Wen and Zeng (2010) and Luo and Zhou (2017) also found that the left-behind experience had different effects on individuals' personalities, emotions, interpersonal relationships, and learning. From the perspective of evolutionary psychology, individuals have to develop strong resilience in order to survive adversity. For example, Science News Staff (2018) found that many animals, plants, and bacteria use different resilience strategies when faced with scarce resources, predators, and other challenges, such as stealing genes or switching sex (Science News Staff, 2018). (Browman et al., 2017) found that despite all the disadvantages, many low socioeconomic status students still maintain high learning motivation and academic persistence. Therefore, resilience improves survival. We also observed significant differences in certain dimensions of psychological capital when considering students' gender, grade, and student leader experience. Gender had a significant predictive effect for self-efficacy, hope, optimism, resilience, and PsyCap. Specifically, the psychological capital, self-efficacy, and resilience scores for boys were significantly higher than those for girls, which is probably due to the stereotype and role orientation of gender in Chinese culture. Liu et al. (2011) and Zuo et al. (2013) concluded that traditional Chinese culture often requires a man to be stronger, more independent and confident, and have a greater sense of family responsibility, while females are supposed to be more delicate, sensitive, and emotional. Thus, boys will receive more support and investment during their growth and education, which helps cultivate a positive attitude and healthy coping behaviors. Therefore, the left-behind experience had little impact on the development of boys' psychological capital and other dimensions. Conversely, girls were prone to be anxious and felt inferior.

USWL who served as student leaders scored higher on all four psychological capital dimensions than those who did not. This variable had significant predictive effects for Hope and total PsyCap. Avolio et al. (2004) proposed that psychological capital can be measured and effectively managed and developed. Our results demonstrate that serving as a leader may promote individual's psychological capital by providing students more social practice and training opportunities that help them develop a more comprehensive and positive understanding of themselves.

USWL in different grades had significant differences in total score of psychological capital, self-efficacy, hope, and resilience, and grade had a significant predictive effect for Hope. Freshmen and sophomores had the highest and lowest psychological capital scores, respectively. This could be explained by the fact that freshmen were free of academic and examination pressure from high school and expected university life to be exciting. In the second year, university life was no longer new to them, and they were faced with academic stress, complex interpersonal relationships, and employment pressure, causing their psychological capital to fall dramatically. In the third year, they had acclimated to university life, and their psychological capital rose again as their participation in interpersonal and social events increased. This is consistent with the “low tide sophomore year” phenomenon proposed by Taylor and Cuave (1994) and Wang and Kennedy-phillips (2013). The second year in university was also a turning point for USWL psychological capital.

This study found that which parent(s) went out working and left-behind duration did not significantly influence USWL psychological capital, self-efficacy, hope, optimism, or resilience. However, a longer stay was associated with lower psychological capital development, which is consistent with previous findings (Marchetti-Mercer, 2012; Lu, 2015; Sun et al., 2017). The self-efficacy, hope, optimism, and total psychological capital of USWL in contact with their parents every 1–2 weeks were significantly higher than those with less frequent contact (annually or less). The scores for self-efficacy, hope, optimism, and psychological capital of USWL who saw their parents monthly were significantly higher than those who reunited annually. However, the latter group had the highest resilience scores. This indicates that the frequencies of parent contact and reunification affect USWL psychological capital. Higher frequencies are associated with greater confidence and optimism, and these individuals may have higher perseverance. Those who see their parents infrequently have higher resilience. Students whose guardians were grandparents scored significantly higher on hope, optimism, and psychological capital than those who had been watched by relatives or neighbors. Grandparents are the closest relatives to USWL except their parents, so they are likely to provide comparable support. USWL cared for by more distant relatives or neighbors may experience feelings of interception and insecurity, so they have lower psychological capital.

### Discussion of Study 2

Pre- and post-test comparisons on psychological capital data revealed that the psychological capital of students in the experiment group was remarkably enhanced after eight psychological group interventions, whereas there was no change in the control group. This indicates that the intervention activity based on PCI theory can effectively improve the psychological capital of USWL. This is consistent with the conclusions of previous studies (Rew et al., 2014; Russo and Stoykova, 2015; Deng et al., 2016).

After the group intervention activity, feedback from the open questions on the questionnaire showed that most students felt they knew more about themselves and were more confident than before. Stress was alleviated, they felt happier, and their attitude was more positive and optimistic. Their problem-solving abilities were enhanced, as were the characteristics of resilience and hope. Levels of self-efficacy, optimistic attitude toward life, and psychological resilience in the face of setbacks were increased in USWL. These findings suggest that our group intervention can improve the psychological capital of university students who were left behind. The group intervention design was mainly based on Luthans' PCI theory, which proposed methods to assess the development of four dimensions: confidence, resilience, hope, and optimism. The utility and effectiveness of this approach were confirmed in Luthans' empirical studies on micro and network interventions (Luthans et al., 2006a). Moreover, the concept of psychological capital counseling is highly suitable for students who were left behind in childhood. Positive psychology can help individuals accept their reality and see themselves in a more positive way. For example, optimistically viewing the left-behind experience may help individuals find advantages that help them establish a better life.

Secondly, we implemented a rational activity plan using the psychological capital theory. The activities fit the theme and were deemed suitable for the students. The intervention was divided into eight themes. The first and second were in the group establishment stage. Members were guided to build a secure community atmosphere. They were informed about the ultimate goal of the activity and asked to adhere to the rules. At the second event, members became more familiar with each other, which strengthened group cohesion. The third to seventh events were based on activity themes with a strong link to improving psychological capital. In the eighth and final event, members shared their feelings of group attachment and dealt with separation anxiety that parting might bring. In order to guarantee coherence, each activity began with a warm-up concluded with a brief review and summary.

Thirdly, due to the uniqueness of the USWL group, students found mutual understanding, which facilitated intimate relationships. Through respect and acceptance, members became more confident in problem solving. It is meaningful for students to experience a harmonious community atmosphere where everyone has free expression and is accepted by others. This environment allows the adoption of a constructive attitude and encourages positive behavior. Interactions among group members also improved effective communication and confidence. Positive and timely feedback helped members adjust their interpersonal mode and build interpersonal skills.

## General Discussion

In summary, this study based on the literature analysis found that the psychological capital of USWL is moderate. Their self-efficacy, optimism, hope, and overall psychological capital are significantly lower than control subjects. Importantly, their psychological resilience is remarkably higher than those who were not left-behind. Our results suggest that some demographic factors such as gender, grade, only-child status, student leadership experience, parental reunion frequency, and relationship with guardians significantly influence the psychological capital of USWL.

Multiple regression analysis revealed that gender, student cadre status, and grade can significantly predict psychological capital, but different left-behind experiences are less predictive. Our results suggest that multiple factors interact to affect the psychological development of USWL. Some people who experience serious danger and/or adversity show normal or even excellent development. Although parents going out to work can be considered an adverse life event for LC, it does not directly affect mental health. There may be other protective factors at work, such as the economic situation; the social support of parents, teachers and peers; and individual coping style. According to survey results, the application of PCI theory can be used to carry out experimental group interventions and develop strategies that enhance self-understanding, self-efficacy, resilience, rational planning, and hope.

The results of our study should be considered in the context of several limitations. First, it only included students in three provincial universities of China. In addition, we employed a cross-sectional design. Future investigations should utilize various research methods to carry out in-depth and systematic research. For example, a longitudinal study with qualitative methods could explore the changing trend of psychological capital among USWL.

There are also some limitations in the intervention experiment. Again, subjects were selected from a subset of universities of Sichuan province. The lack of representativeness and small sample size could affect result reliability and validity. It is necessary to further expand the scope to include subjects from diverse family backgrounds. We focused on subjects from rural areas, but students whose parents went to work abroad could also be considered as LC under the changes to China's economic development policy. Second, the intervention length was not sufficient. There were eight 1.5-h group intervention sessions. Psychological capital is a state that can be affected by managing and developing different variables; the overall development of psychological capital, resilience, hope, optimism, and self-efficacy is a continuous, lengthy process. Short-term interventions have limitations. It would be appropriate to increase the number/length of interventions and employ longer follow-up (e.g., 3, 6, or 12 months) in future studies. Third, although it was supported by related theories, the group intervention of this study remains to validated. For example, group members' incomplete sharing was likely affected by inaccurate instruction. In addition, it is unclear whether the effect of psychological capital group intervention is persistent. We found that PCI played a significant role in improving the psychological capital of USWL, but the result was collected after the intervention. Additional investigations are needed to clarify the continuity and delay effects of our intervention.

## Conclusion

We studied students from three universities in Sichuan province who had left-behind experiences in childhood. We found that the psychological capital of USWL is moderate, with lower self-efficacy, optimism, hope, and overall psychological capital than control subjects. However, their psychological resilience is remarkably higher than those who were not left-behind. Secondly, some demographic factors such as gender, grade, only child status, student leadership experience, reunion frequency with parents, and the relationship with guardians significantly influence the psychological capital of USWL. Thirdly, a group intervention based on the theory of PCI can effectively improve the psychological capital of USWL.

## Ethics Statement

This study was carried out in accordance with the recommendations based on the guidelines by the Ethics Committees of Southwest Petroleum University, Sichuan Normal University, Xihua University in Sichuan Province in China. Written informed consent was obtained from all subjects.

## Author Contributions

LL was the primary investigator, provided comments and ideas, and wrote and revised the manuscript. QX helped design the study and revise the manuscript. YY provided comments and ideas, and helped edit the manuscript.

### Conflict of Interest Statement

The authors declare that the research was conducted in the absence of any commercial or financial relationships that could be construed as a potential conflict of interest.
